# Charge density mapping in a pediatric patient with symptomatic runs of ectopic atrial tachycardia

**DOI:** 10.1016/j.hrcr.2024.03.012

**Published:** 2024-04-01

**Authors:** Sip A. Wijchers, Gert van den Berg, Janneke A.E. Kammeraad, Thomas B. Krasemann, Sing-Chien Yap

**Affiliations:** ∗ Department of Cardiology, Thoraxcenter, Cardiovascular Institute, Erasmus MC, Rotterdam, The Netherlands; † Department of Pediatric Cardiology, Erasmus MC – Sophia Children’s Hospital, Rotterdam, The Netherlands

**Keywords:** Charge density mapping, Atrial tachycardia, Premature atrial complexes, Pediatric, Noncontact mapping

**Key Teaching Points**

• Single-beat mapping provides a new mapping technique for nonsustained atrial tachycardia in the pediatric population.
• With careful measurements of venous and intracardiac diameters, the use of large-bore sheaths and basket catheters is feasible.
• Charge density mapping offers possibilities for a broader spectrum of arrhythmias to be mapped in the pediatric population.

## Introduction

Ectopic atrial tachycardia (AT) poses a notable challenge in pediatric cardiology, often requiring precise localization for optimal treatment. Its incidence is relatively low, affecting 3.7%–5.7% of children undergoing electrophysiological studies.^1^ While catheter ablation complements pharmacological therapy, achieving a high success rate of approximately 90%, there is room for enhancement.^1^ A challenge lies in mapping and ablating transient episodes of AT. Recent innovations in mapping technologies, notably charge density mapping, have shown promise in providing precise single-beat noncontact mapping of transient AT in adult patients.^2^^,^^3^ This mapping technique uses individualized anatomy of a heart chamber, created by ultrasound crystals that are integrated in a basket catheter, with subsequent visualization of recorded cardiac dipoles.^4^ This article explores a unique case with short runs of ectopic AT, demonstrating the efficacy of single-beat noncontact mapping using charge density mapping in a pediatric patient.

## Case report

**Figure 1:**
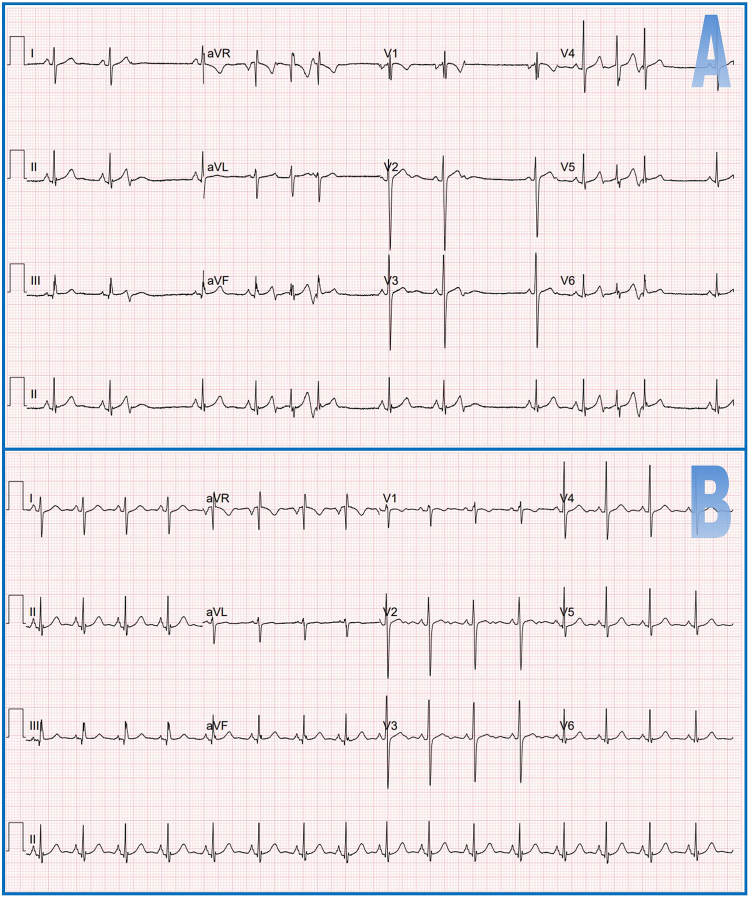
Electrocardiogram before the procedure (**A**) and at a 3-month interval after the procedure (**B**).

A 10-year-old boy, weighing 48.9 kg, presented with symptomatic episodes of AT. His baseline electrocardiogram (ECG) is shown in Figure 1A. Holter monitoring demonstrated a substantial burden of premature atrial complexes (PACs) and ATs, accounting for 32% of cardiac cycles. Echocardiography confirmed a structurally and functionally normal heart. Given the patient’s inadequate response to antiarrhythmic medication, he was scheduled for catheter ablation of his PACs. The right atrial diameter exceeded 25 mm, thus permitting the introduction of a multipolar basket catheter with a deployed diameter of 25 mm and with 48 integrated ultrasound transducers and 48 high-fidelity, low-impedance electrodes (AcQMap; Acutus Medical, Carlsbad, CA).

**Figure 2:**
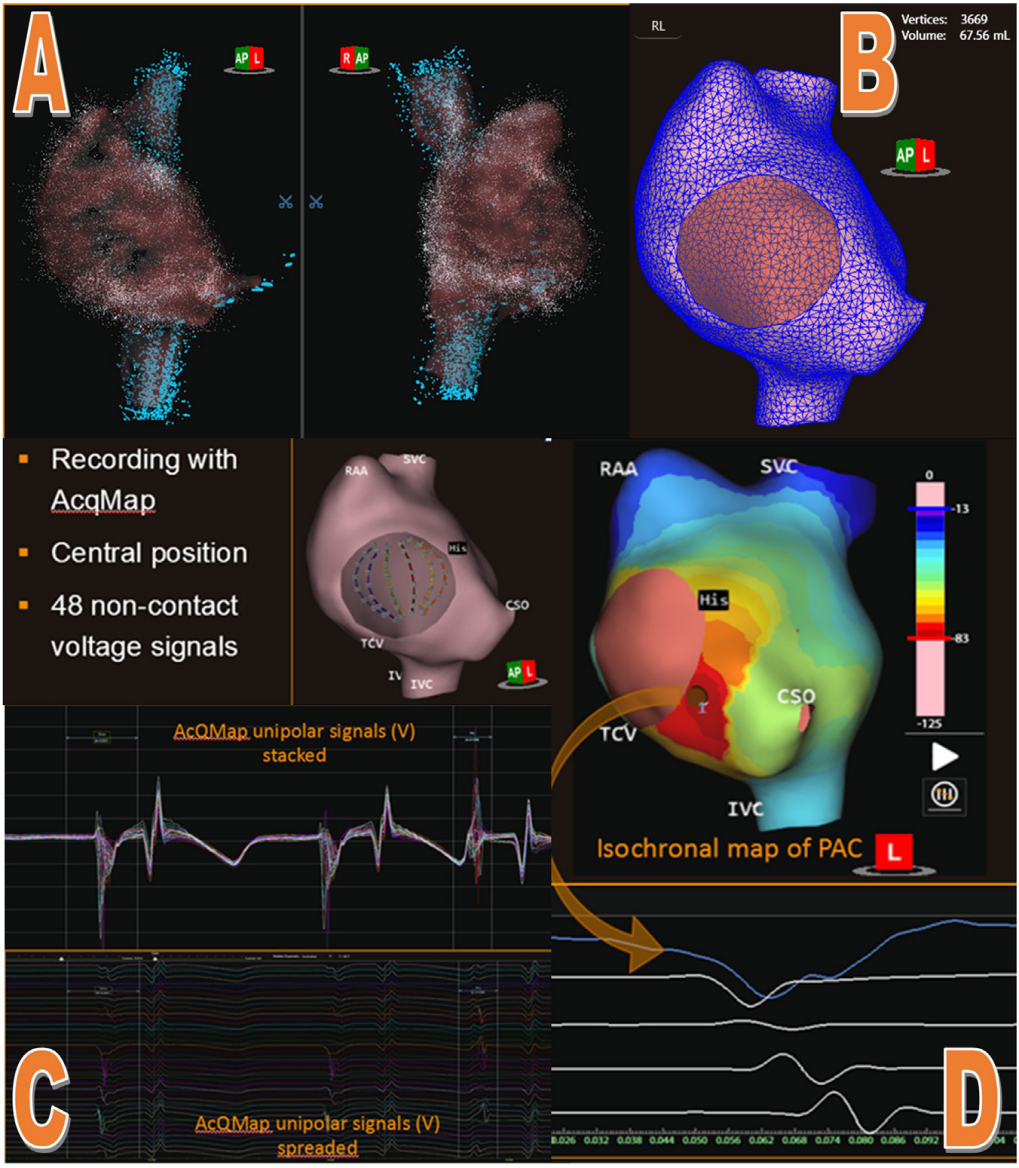
Creation of the right atrial activation map (AcQMap; Acutus Medical, Carlsbad, CA). **A:** Creation of the ultrasound point cloud. **B:** The resulting mesh network. **C:** Acquisition of signals. **D:** The resulting activation map together with the local signals at the site of earliest activation (*blue line and orange arrow*). CSO = coronary sinus ostium; IVC = inferior vena cava; PAC = premature atrial complex; RAA = right atrial appendage; SVC = superior vena cava; TCV = tricuspid valve.

**Figure 3:**
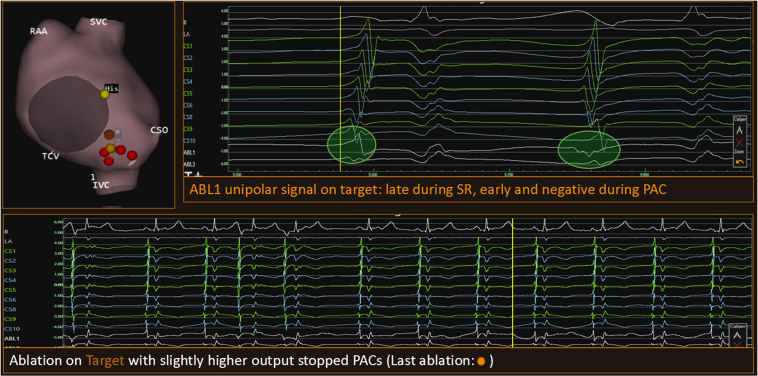
The location and the signals of the site of successful application. CSO = coronary sinus ostium; IVC = inferior vena cava; PAC = premature atrial complex; RAA = right atrial appendage; SR = sinus rhythm; SVC = superior vena cava; TCV = tricuspid valve.

The procedure was conducted under general anesthesia. Vascular access was established using Seldinger’s technique, guided by ultrasound, in both groins. The vascular diameter was measured before inserting the sheaths. A 12.4F (15.2F outer diameter) steerable long sheath (AcQGuide; Acutus Medical) was positioned in the right femoral vein, through which the basket catheter was introduced into the right atrium. Additionally, an 8.5F sheath was introduced in the left femoral vein to accommodate the remote magnetic navigation ablation catheter (MagnoFlush; MedFact, Lörrach, Germany), while a 7F (10.8F outer diameter) sheath with integrated reference electrodes (AcQRef; Acutus Medical) facilitated the placement of the decapolar coronary sinus catheter. An activated clotting time longer than 350 seconds was achieved with heparin. The target activated clotting time is >350 seconds. The aforementioned multipolar basket catheter was introduced into the right atrium over the wire. The subsequent steps, illustrated in Figure 2, outlined the creation of a noncontact charged-density map. A spherical basket catheter is introduced into the right atrium and by using ultrasound creates an anatomical model of the right atrium. This model, along with unipolar measurements from the basket’s electrodes, creates an electroanatomical activation map, which can be derived from a single beat. Charge density mapping effectively replicated the electrical field of the atrial activation.^4^ The AcQMap revealed the earliest activation of the ectopic atrial activity just below the coronary sinus ostium, as depicted in Figure 3. Multiple propagation-history maps of multiple PACs during single-position mapping were analyzed to confirm spatial stability of the ectopic focus. They all showed the exact same point of origin. For single-position maps, the AcQMap catheter needs to be centrally positioned within the chamber of interest. The unipolar voltages together with the endocardial anatomy are key inputs for the inverse solution to derive the location of charge sources on the endocardial surface

A series of 6 radiofrequency applications were administered at the point of earliest activation, with the final application closest to the site defined by AcQMap. The last application resulted in complete elimination of ectopic activity. During the following 30 minutes no further ectopic atrial activity was seen. Vascular closure was achieved through manual compression. Importantly, no vascular or procedural complications were encountered. The procedure time was 136 minutes (time from groin puncture until removal of all sheaths, including 30 minutes waiting time), fluoroscopy time was 6.3 minutes, and total dose-area product was 201 cGy·cm^2^. The postprocedural ECG is shown in Figure 1B.

Holter monitoring at 3 months postablation demonstrated the absence of both AT and PACs.

## Discussion

This case proves that single-beat noncontact mapping using charge density mapping is suitable for pediatric patients. The utilization of multiple electrodes capable of recording cardiac dipole charges without direct wall contact enables the mapping of even single ectopic beats to their origin. Given that children are typically treated under general anesthesia, and at least part of these arrhythmias may be sensitive to adrenergic stimulation, there is a likelihood of a significantly reduced arrhythmia burden during the procedure.^5^ We like to emphasize that appropriate patient selection in the pediatric population is important, considering the use of large-bore sheaths and basket dimensions. We recommend that femoral vein dimensions should be appropriate to accommodate a 15.2F sheath. Furthermore, the right atrial dimension should be larger than 25 mm to accommodate full deployment of the basket catheter without contact of the atrial wall. The application of AcQMap, with careful consideration of right atrial dimensions and femoral vein size, was safe and effective in mapping and ablating this ectopic AT. Though the steerable sheath’s size poses a limitation for small children, meticulous measurement of the femoral vein and guided access by ultrasound presented no issues in this case. Furthermore, hemostasis was easily achieved through manual compression. This way of mapping allowed precise localization of the nonsustained AT, which exhibited a diminished burden under general anesthesia. In this case the preprocedural burden of atrial ectopy was high (32%) during Holter monitoring. Furthermore, the preprocedural ECG showed bursts of atrial ectopy with a single or double PAC after every other sinus beat. After the start of anesthesia there was a significant reduction in the burden of atrial ectopy to an occasional PAC every so many sinus beats.

Mapping infrequent bursts of AT or sporadic PACs can be quite challenging with traditional 3-dimensional location-based mapping systems owing to their limited capability. However, given this limitation and the specific case described, there is a clear need for single-beat mapping. The recent advancement of mapping systems also highlights the importance of single-beat mapping. For instance the VIVO system (Catheter Precision, Inc, Mt Olive, NJ) demonstrates the significance of single-beat mapping in mapping PVCs through skin electrodes.^6^ Both of these systems were developed to accurately locate sporadic ectopy more efficiently and reliably.

Unfortunately, shortly after the case described in this study, the Acutus system ceased production of the catheter and discontinued support for their software. While originally developed for mapping sources and rotors in atrial fibrillation, its true strength, in our opinion, lay in single-beat noncontact mapping of short-lived ATs. Considering all of the aforementioned points, it is evident that there is a need for a system like this. However, we have concerns that the niche market for such a system may be too small for a new system to emerge in the near future.

Single-beat mapping holds the promise of refined management strategies for pediatric arrhythmias, particularly in cases where pinpointing the arrhythmia’s exact origin proves elusive. Notably, in the adult population, the aforementioned Acutus system has successfully mapped intra-atrial reentry tachycardia and complex accessory pathways, with a potential for the pediatric population as well.^7^, ^8^, ^9^ With further experience, a broader array of arrhythmias in the pediatric population might benefit from the individualized anatomy and high precision, especially in short-lived tachycardias or complex arrhythmia circuits. Faster mapping and ablation in these cases may have positive implications for the safety profile of complex pediatric ablations.

## Conclusion

The presented case represents a notable advancement in the field of pediatric cardiac ablation. The successful application of AcQMap in mapping and ablating ectopic AT offers a promising alternative technique for young patients with arrhythmias that were previously challenging to treat effectively, owing to either the nonsustained character or the complex activation during the tachycardia. This case report proves that the use of large-bore sheaths is safe in the pediatric population if careful measurements of venous and cardiac diameters are carried out in preparation for the procedure.

## Disclosures

SAW has received a speaker fee from Biotronik and Daiichi Sankyo. GB has no conflicts of interest. JAEK has received a research grant from Medtronic. TBK has received a speaker fee from Abbott. SCY has received honoraria from Boston Scientific, Medtronic, Biotronik, and Acutus Medical; and research grants from Medtronic, Biotronik, and Boston Scientific.
